# A novel compound heterozygous mutation of the *CLCN7* gene is associated with autosomal recessive osteopetrosis

**DOI:** 10.3389/fped.2023.978879

**Published:** 2023-04-24

**Authors:** Xia Wang, Yingcan Wang, Ting Xu, Yanjie Fan, Yifeng Ding, Jihong Qian

**Affiliations:** ^1^ Department of Neonatology, Xinhua Hospital Affiliated to Shanghai Jiaotong University School of Medicine, Shanghai, China; ^2^ Department of Pediatric Endocrinology/Genetics, Xinhua Hospital Affiliated to Shanghai Jiaotong University School of Medicine, Shanghai, China; ^3^ Department of Neurology, Children's Hospital of Fudan University & National Children Medical Center, Shanghai, China

**Keywords:** autosomal recessive osteopetrosis (ARO), chloride channel 7 (*CLCN7*), leukocytosis, neurological impairment, genotype–phenotype correlation

## Abstract

Osteopetrosis is a genetic condition of the skeleton characterized by increased bone density caused by osteoclast formation and function defects. Osteopetrosis is inherited in the form of autosomal dominant and autosomal recessive manner. We report autosomal recessive osteopetrosis (ARO; OMIM 611490) in a Chinese case with a history of scarce leukocytosis, vision and hearing loss, frequent seizures, and severe intellectual and motor disability. Whole-exome sequencing (WES) followed by Sanger sequencing revealed novel compound heterozygous mutations in the chloride channel 7 (*CLCN7*) gene [c.982-1G > C and c.1208G > A (p. Arg403Gln)] in the affected individual, and subsequent familial segregation showed that each parent had transmitted a mutation. Our results confirmed that mutations in the *CLCN7* gene caused ARO in a Chinese family. Additionally, our study expanded the clinical and allelic spectrum of the *CLCN7* gene and enhanced the applications of WES technology in determining the etiology of prenatal diagnoses in fetuses with ultrasound anomalies.

## Introduction

Osteopetrosis is a genetically heterogeneous disorder characterized by abnormal bone metabolism. The pathogenesis of osteopetrosis stems from the dysfunction of differentiation and/or absorption of osteoclasts, which results in skeletal dysplasia, such as increased bone density and medullary cavity stenosis (1). Three clinical types can be identified based on severity, age of onset, and inheritance: a dominant benign type, autosomal dominant osteopetrosis (ADO, OMIM 166600); intermediate autosomal osteopetrosis (IAO, OMIM 259710); and a severe recessive type, autosomal recessive osteopetrosis (ARO, OMIM 611490). ARO, also known as infantile malignant osteopetrosis, is the most fatal type of osteopetrosis, with an incidence of 1/250,000 live births. ARO patients usually present by 2 years of age and die before 10 years of age (1–3). Patients diagnosed with ARO are more susceptible to hematological impairment with anemia, thrombocytopenia, and secondary neurological deficits. However, leucocyte counts in ARO patients were not frequently reported (4).

At least 10 genes have been reported to be associated with osteopetrosis*.* Mutations in T-cell immune regulator 1 (*TCIRG1*) and chloride channel 7 (*CLCN7*) are the two most common causes of ARO. *TCIRG1* is located on chromosome 11q13 and spans ∼12.5 kb. The transcript variants of this gene containing 15 exons represent the a3 subunit of the vacuolar proton pump, which is preferentially expressed in osteoclasts and plays an important role in bone resorption. *TCIRG1* mutations are reportedly responsible for over 50% of ARO cases. The *CLCN7* gene on human chromosome 16p13.3 contains 25 exons and encodes the 803 amino acid chloride channel protein 7 (CLC-7). *CLCN7* is a member of the voltage-gated chloride channel protein family that mediates the exchange of chloride ions against protons, maintaining the acidic environment for bone resorption (5). *CLCN7* plays a synergistic role when hydrogen ions are transported outside of the cell by *TCIRG1* (6). Mutations in *CLCN7* have been documented in approximately 20% of individuals and are related to a broad spectrum of osteopetrosis with phenotypes ranging from mild to life-threatening levels of severity (1, 3, 7).

The initial clinical presentation of this rare genetic syndrome is heterogeneous. Formal clinical diagnostic criteria for ARO have not been established. High awareness of initial ARO symptoms is critical for early diagnosis. Approximately 100 variants in *CLCN7* are known (http://www.hgmd.cf.ac.uk/). ARO related to *CLCN7* mutation has been identified in approximately two dozen families (3, 8–19). Affected individuals admitted for visual impairment, anemia, failure to thrive, or convulsion in the infantile period are frequently reported (1, 3). These symptoms and radiological changes involving extensive bone calcification should raise clinical suspicion of ARO. Genetic testing approaches promote molecular diagnoses. Data regarding Chinese patients with *CLCN7*-related ARO are limited to four cases from Taiwan (12), Guangzhou (10), and Shanghai (17). These patients had a relatively stable disease course and were still alive at the time they were reported.

Here, we report the natural course of a patient with *CLCN7*-related ARO involving scarcely leukocytosis at birth, rapidly progressing to neurological deterioration with a very poor prognosis. We also review the clinical and genetic findings in *CLCN7*-related ARO reported thus far.

## Methods

### Subjects and ethical approval

A neonate who presented with severe leukocytosis and thrombocytopenia at 3 days of age was referred to Xinhua Hospital affiliated with Shanghai Jiao Tong University School of Medicine. The patient was evaluated with whole-exome sequencing (WES) for etiological evidence.

The studies involving human participants were reviewed and approved by the Institutional Review Board of Xinhua Hospital*.* Written informed consent was obtained from the legal guardian for the publication of any potentially identifiable images or data included in this article.

### Whole-exome sequencing and Sanger sequencing

Genomic DNA was extracted from peripheral blood. Library preparation was performed with an xGen Exome research panel v1.0 (IDT, United States), and sequencing was conducted on a HiSeq 4000 (Illumina, United States). Raw reads were aligned to the reference genome GRCh37/hg19 by BWA-MEM (v0.7.12). Variant calling was performed following the GATK best practice workflow (v3.3) (20, 21). Variant annotation and filtration were performed by SnpEff (v4.2) and SnpSift (v4.2) based on gnomAD (v2.1), OMIM (https://omim.org/), HGMD (https://www.hgmd.cf.ac.uk/ac/), ClinVar (https://www.ncbi.nlm.nih.gov/clinvar), and an in-house database. Interpretation of variants was conducted in accordance with American College of Medical Genetics and Genomics (ACMG) guidelines (22).

The variant screening process was based on disease-related information and variant pathogenicity evaluation (allele frequency in population, *in silico* tools, ACMG guidelines) (23, 24). Genetic disorders that frequently manifest phenotypes similar to our case include juvenile-myelomonocytic-leukemia (JMML) (5, 17, 25) and leukocyte adhesion deficiency (LAD) (1, 26, 27). JMML-related genes include *PTPN11*, *NRAS*, *KRAS*, *NF1*, and *CBL*. LAD-related genes include *IKBKG*, *ITBG*, *KINDLIN3*, and *FERMT3.* All rare variants in these genes were extracted and filtered.

Sanger sequencing was performed to validate the heterozygous variants identified through WES. PCR amplification was carried out using an ABI 9700 Thermal Cycler and sequenced on an ABI PRISM 3730 sequencer (Applied Biosystems, United States) using the primers listed in Supplementary Table S1.

### *In silico* analysis

REVEL is a tool for predicting the effect of reported missense variants on the function of mutant human proteins (28). The chromosome localization, protein sequences, and phylogenetic tree of the missense variant (c.1208 G > A) in the *CLCN7* gene were analyzed by ClustalX_1.81 (http://www.clustal.org/) and Molecular Evolutionary Genetics Analysis (https://www.megasoftware.net/). Mutant sequences (c.982-1G > C) and wild-type sequences were analyzed by *in silico* tools (29), CADD (Combined Annotation Dependent Depletion; cadd.gs.washington.edu) splice, MaxEntScan, Spliceogen (https://github.com/VCCRI/Spliceogen), NNSPLICE (Splice Site Prediction by Neural Network), and Splice AI (https://github.com/Illumina/SpliceAI).

## Results

### Clinical presentation

**Figure 1:**
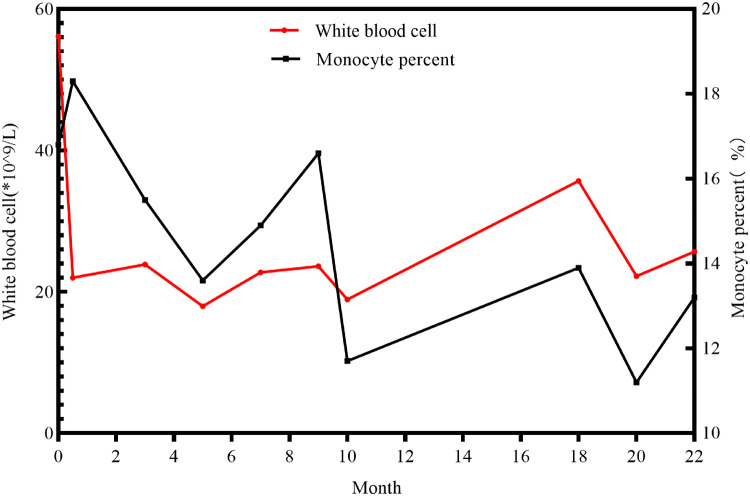
The longitude data of white blood cells and monocyte percent of the patient.

**Figure 2:**
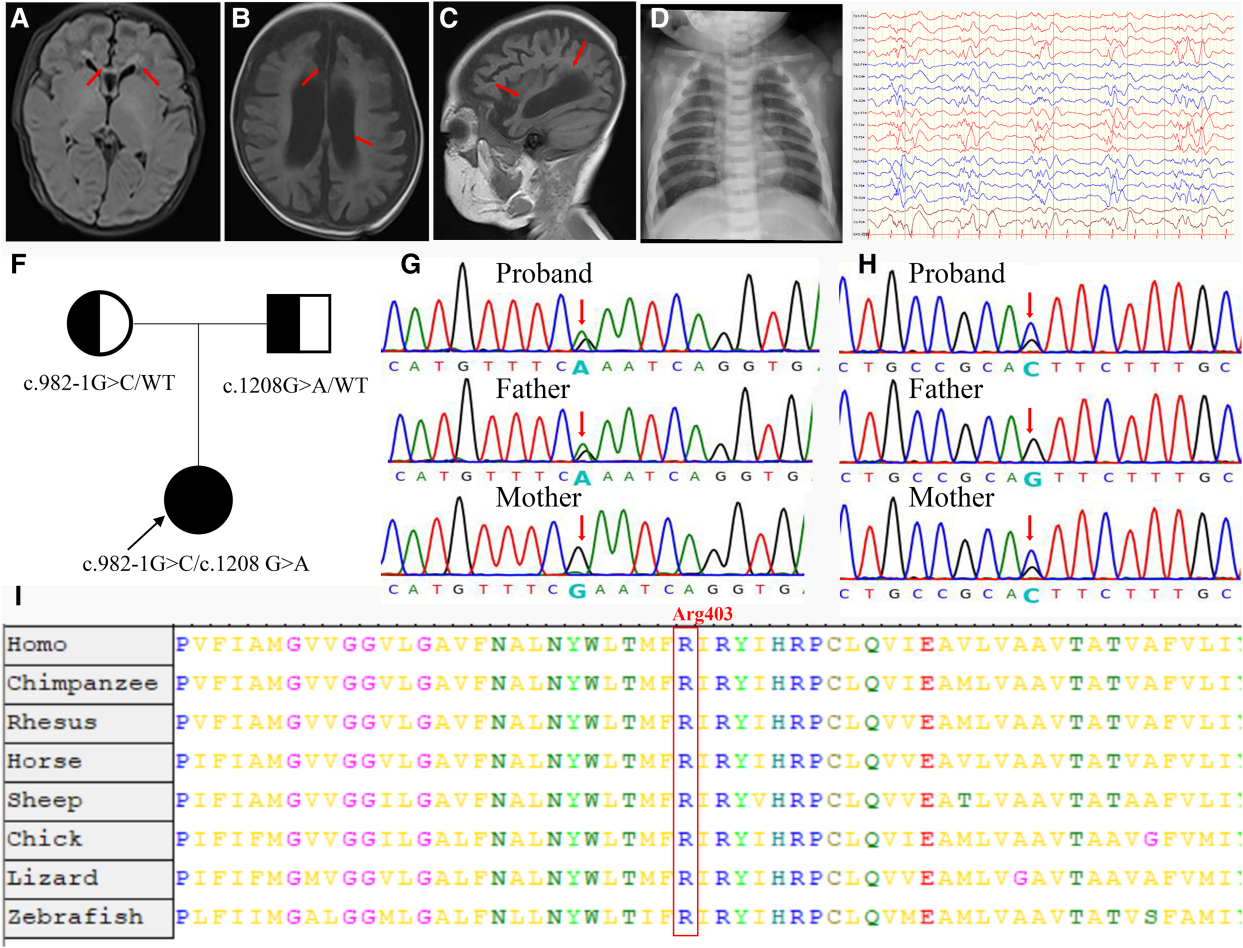
Clinical and molecular findings of the patient. (**A**) T1-weighted (T1W) brain MRI indicated a high signal in the white matter (arrowheads) and corpus callosum (arrowheads) during the neonatal period. (**B,C**) T1W brain MRI indicated hydrocephaly (arrowheads) and brain atrophy (arrowheads) at 4 months of age. (**D**) Proband showed increased bone density on radiography and “sandwich” vertebrae. (**E**) Multifocal slow spike-wave and slow-wave patterns in electroencephalogram. (**F**) The proband's family tree. (**G**) The proband was paternally inherited the variant of c.1208G > A in the *CLCN7* gene. (**H**) The proband was maternally inherited the variant of c.982-1G > C in the *CLCN7* gene. (**I**) A cross-species alignment of amino acid sequences showed that *p*. Arg403Gln variants were located in a highly conserved region in CLCN7 protein.

A 3-day-old female neonate was referred to our hospital with rare leukocytosis (56.12 × 10^9^/L) and transient thrombocytopenia (85 × 10^9^/L) on the day she was born. Physical examination showed no external birth defects other than mild hypotonia with slow suckling. Clinical biochemical analysis was also performed. The patient's hematological profile was as follows: white blood cell (WBC) count, 43.71 × 10^9^/L; neutrophil/lymphocyte (N/L) ratio, 66%/16.9%; monocyte ratio, 14.70%; hemoglobin (Hb), 162 mg/dL; platelets, 98 × 10^9^/L. C-reactive protein and procalcitonin were in the normal range. No pathogenic microorganisms were identified by bacterial culture or metagenomic sequencing. Serology for toxoplasma, rubella, cytomegalovirus, and herpes was negative. Peripheral blood smear showed no evidence of immature cells. Bone marrow puncture was considered but not successfully performed. After intravenous administration of meropenem and penicillin for 7 days, leukocytosis and thrombocytopenia did not significantly improve (Figure 1). Brain magnetic resonance imaging (MRI) showed slight signal alterations in white matter and the corpus callosum (Figure 2A). Screening tests of hearing and ocular disease were normal.

At 2 months of age, the patient had vision and hearing impairment, with little response to colors, voices, or moving objects. From the age of 4 months, her neurological defects gradually worsened. The patient began to have seizures that manifested as spasticity, sometimes as often as dozens of times per day, and a diagnosis of West syndrome was made. She was treated with various antiepileptic medicines, such as vigabatrin, topiramate, and levetiracetam, but none successfully controlled the seizures. The patient's biochemical indexes were normal, including calcium–phosphorus metabolism biomarkers, immunoglobulin levels, and lymphocyte classification counts (Supplementary Table S2). Brain MRI showed hydrocephalus and cerebral atrophy (Figures 2B,C). The electroencephalogram showed multifocal slow spike-wave and slow-wave patterns (Figure 2E). At 9 months of age, her head circumference was 38.0 cm (<−3 SD), height was 65 cm (<−2 SD), and weight was 7.0 kg (−1 SD to −2 SD). Her vision was completely lost. She could not raise her head, sit, or laugh. Neuropsychological testing showed a development delay using the Bayley III scale.

Through telephone follow-up, we learned that her leucocytes remained at a high level (not lower than 15 × 10^9^/L). When the patient experienced diarrhea or acute upper respiratory infections, her leucocytes were high, up to 20–40 × 10^9^/L (Figure 1). Because of the progressive neurologic impairment, her family was compelled to provide symptomatic treatment without resorting to bone marrow transplantation or hematopoietic stem cell transplantation. The patient died from respiratory arrest at the age of 22 months.

### Molecular analysis

Compound heterozygous variants were identified in the *CLCN7* gene. According to ACMG guidelines, the variant c.1208G > A (p. Arg403Gln) is rated “likely pathogenic” (PM1 + PM2 + PM3 + PP3 + PP4). The variant c.982-1G > C, located at a splice donor site, is also rated “likely pathogenic” by ACMG standards (PVS1_S + PM2 + PM3 + PP4).

We also reviewed the patient's bone imaging, which showed increased bone density on radiography and “sandwich” vertebrae (Figure 2D); this evidence, together with the early onset of patient clinical manifestations and the mode of inheritance (Figure 2F), was highly compatible with ARO. The variant c.1208G > A was paternally inherited (Figure 2G). This missense mutation is a previously reported osteopetrosis-causing variant located in the transmembrane domain of the protein. The c.1208G > A mutation is located in a highly conserved region among vertebrates, as confirmed by an online sequence database (Figure 2I). The other variant, c.982-1G > C, located in intron 11, was maternally inherited; this variant is novel (Figure 2H).

### Splice-altering prediction

As a variant at the canonical splice site, the interpretation of the c.982-1G > C variant following ACMG guideline was “likely pathogenic” (PVS1_S + PM2 + PM3 + PP4). In order to predict the possible splice-altering effect, multiple splice-altering prediction algorithms were used including CADD, MaxEntScan, Spliceogen, NNSPLICE, and SpliceAI.

The guanine is replaced by thymine (c.982-1G > C) at nucleotide number 1 in intron 11 located at the exon–intron boundaries. *In silico* prediction analysis is shown in Table 1. The CADD index is 32 (CADD index of process normal order is 14.25). MaxENT value is 8.27 (MaxENT index of the normal sequence is 2.35). Spliceogen index was 1.0 (normal sequence index is 0.78). NNSPLICE showed an original acceptor site with a score of 0.79 (cutoff 0.40); no splice site was predicted after the c.982-1G > C alteration. Similarly, SpliceAI suggested splice acceptor loss caused by the variant, with a score of 0.79 (cutoff 0.50). Based on these prediction algorithms, the c.982-1G > C variant, at the splice acceptor site of intron 11, potentially alters mRNA transcription.

**Table 1:** Splicing prediction of the novel variant c.982-1G > C in *CLCN7* gene.

In silico tools	NM_001287.5: c.982-1G > C		Splice site altering
	Scores	Cutoff	
CADD splice	32	>14.25	Yes
MaxEntScan	8.27	>2.35	Yes
Spliceogen	1.0	0.78	Yes
NNSPLICE	0.75	>0.4	Yes
SpliceAI	0.79	>0.1	Yes

CADD splice, combined annotation-dependent depletion splice; NNSPLICE, splice site prediction by neural network.

## Discussion

We reported a case of neonatal *CLCN7*-related ARO detected at birth, with an onset symptom of severe leukocytosis; later symptoms included vision and hearing loss as well as neurological deficits in the form of frequent seizures and intellectual and motor disability. The diagnosis was confirmed by genetic testing, which showed compound heterozygous variants in the *CLCN7* gene.

Anemia and thrombocytopenia are prominent symptoms in ARO patients due to bone marrow failure (Table 2). Recurrent leukocytosis observed in our patient has been scarcely reported. Another case of *CLCN7*-related ARO reported in Guangzhou, China, showed an increased leukocyte count of 19.2 × 10^9/L at 7 months of age, without longitudinal data, which was similar to our patient's condition. However, studies of immunological deficits in ARO patients have been limited by the availability of osteoclasts. The defective generation of superoxide by neutrophil cells, monocytes, and lymphocytes, which results in an inability to eradicate infection, has been observed in ARO patients (31, 32). Recurrent leukocytosis and osteopetrosis at early age was highly suspected to be due to *KINDLIN3* mutation, an important intracellular signaling molecule involved in the combination of osteoclast maturation and leukocyte adhesion deficiency (26, 33). However, genomic sequencing did not identify pathogenic variants. Second, the monocyte percentage in this case was persistently increased (Figure 1). Monocyte macrophages and osteoclasts are derived from the same hematopoietic lineage. The dysfunction of osteoclasts may trigger an increase in the production of monocytes in the feedback cycle, which is modulated by homologous crosstalk signals (7, 34). Furthermore, determining whether leukocytosis is a phenotype in *CLCN7*-related ARO children and identifying the underlying genotype may require a more specific cohort.

**Table 2:** Clinical findings from our patient and others with *CLCN7*-related ARO in the literature.

Case (ref.)	Country	Onset age	Onset symptom	Onset blood profile			CNS involvement	Vision loss	Other symptoms	Outcomes
				WBCs (10^9^/L)	Hb (g/L)	Platelets (10^9^/L)				
1 (10)	China	7 months	Epilepsy attacks	19.2	75	71	Epileptic attacks, intellectual disability	Yes	No	Alive
2 (12)	China (Taiwan)	2 months	Poor feeding habits and frequent, irritable crying	9.2	92	131	Poor feeding, hydrocephalus, developmental delay	No	No	Alive
3 (13)	Germany	1 year	Complex neurogenic developmental disorder	NA	NA	NA	Epileptic attacks, macrocephaly	No	No	Dead
4 (14)	Thailand	3 months	Hepatosplenomegaly and anemia	NA	134	NA	No	Yes	No	Alive, 25 years old
5 (11)	Turkey	3 months	Blindness	5.5	109	300	Hypotonicity, intellectual and motor disability	No	Retinal atrophy	Alive
6 (8)	Italy	16 months	A small left wrist fracture	Normal	Normal	Normal	No	No	No	Alive
7a/7b (15)	Jordan	Birth	Severe anemia	Decreased	Severe anemia	Decreased	No	Yes	Hypocalcemia, chronic diarrhea	Dead at 6 months
8a/8b (30)	Japan	14 days	Poor suckling and irritability	NA	NA	Decreased	Epileptic attacks, cerebral atrophy	No	Sepsis arrest	Dead at 19 months
9 (9)	Turkey	10 days	Hypotonicity	14	107	49	Tonic seizure, neurodegenerative disease	Yes	Hypocalcemia; cytomegalovirus infection	Dead at 10 months
10 (3)	NA	3 months	NA	NA	NA	NA	Cerebral atrophy	Yes	…	Dead at 9 months
11 (3)	NA	3 months	NA	NA	NA	NA	Cerebral atrophy	Yes	…	Alive
12 (3)	NA	4 months	NA	NA	NA	NA	Cerebral atrophy	Yes	…	Dead at 1 year
13 (3)	NA	5 months	NA	NA	NA	NA	Macrocephaly	Yes	…	Dead at 4 years
14 (3)	NA	12 months	NA	NA	NA	NA	Autism	Yes	…	Alive
15 (3)	NA	6 months	NA	NA	NA	NA	Macrocephaly, hydrocephalus	No	…	Dead at 1 year
16a/16b (3)	NA	16 months	NA	NA	NA	NA	No	Yes		Alive
17 (17)	China	4 years 7 months	Osteomyelitis, strabismus, mild anemia	9.43	97	204	No	Yes	No	Alive
18 (17)	China	1 year 10 months	Infectious disease with a cold and a cough	8.95	97	194	Hypotonia, severely delayed of motor development milestones	Yes	No	Alive
19 (18)	Pakistani	7 days	Episodes	NA	104	NA	Dysmorphic facies, brain atrophy	Yes	Preterm	Dead at 12 months
20 (19)	Pakistani	Infantile	Difficulty in chewing food	Slightly low	Slightly low	Slightly low	Delayed growth milestones	Yes	NA	Dead at 3 years
21 (16)	Australian	5 years	Fragility fracture	Normal	Normal	Normal	No	No	No	Alive
Present^a^	China	Birth	High leukocyte count	43.71	162	98	West syndrome, intellectual and motor disability, hydrocephalus, atrophy	Yes	No	Dead at 22 months

*CLCN7*, chloride channel 7; ARO, autosomal recessive osteopetrosis; WBCs, white blood cells; Hb, hemoglobin; CNS, central nervous system; NA, not applicable.

^a^ Current study.

Another important issue is central nervous system (CNS) impairment. The mechanism of CNS impairments may result from endosome or lysosome dysfunction in the neuron system mediated by the inactive *CLCN7* channel (35). The interaction pathways of the peroxisome proliferator-activated receptor and neuroactive ligand–receptor are reported to be involved (36). We reviewed the literature and identified a total of 21 patients with *CLCN7-*related ARO (8–17, 30, 37) (Table 2). A total of 15/21 patients had CNS involvement; 6/21 patients died before the age of 5 years. However, the 10/15 patients without CNS involvement were all alive when their cases were reported. The oldest surviving patient was 25 years old (14). Thus, patients with CNS impairments usually had significantly reduced lifespans, and this effect could not be reversed by bone marrow transplantation or hematopoietic stem cell transplantation (1, 3). Thus, CNS involvement in *CLCN7*-related ARO should be thoroughly assessed initially and carefully monitored, as it is an essential prognostic predictor. The typical clinical neurological presentation of *TCIRG1*-related ARO patients was also hydrocephalus, myotonia, neuropsychic and psychomotor retardation, compromised vision, and heavy nasal breathing. Overall, the neurological phenotype of *TCIRG1*-related ARO seems to be more benign than that of the *CLCN7*-dependent form. However, no significant correlation was found between CNS phenotypic presenting features and specific gene mutations, which may be related to the clinical phenotype variability and variable genetic expressivity in the pathogenic genes (4, 38–40).

**Figure 3:**
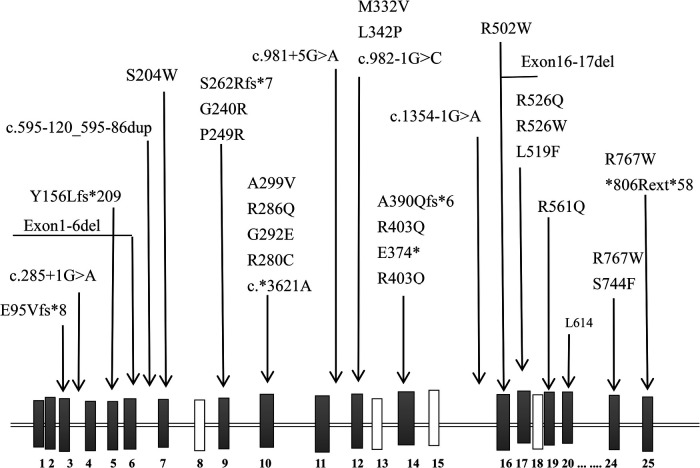
A sketch-map of the gene and location of reported variants of *CLCN7*.

Previously, the reported ARO-causing variants in *CLCN7* (42 alleles, including 22 missenses, 6 splice sites, 3 nonsense, and 5 frameshift) did not include any hotspot mutations (Table 3, Figure 3). The missense mutation (c.1208G > A) was reported, which was suggestive of a mild case of ARO (13). However, our patient's medical history and short survival time manifested a malignant phenotype. Thus, the correlation between genotype and phenotype remains to be resolved. The pathogenetic process may also involve posttranslational modifications, including epigenetic modification, phosphorylation, and ubiquitylation, which might impair the function of the CLCN7 protein.

**Table 3:** The molecular variants in *CLCN7* in our patient and others in the literature.

Case (ref.)	cDNA nucleotide change	Variant type	Heterozygosity	Amino acid change
1 (10)	c.896C > T c.285 + 1G > A	Missense Splice site	Compound heterozygous	p.Ala299Val NA
2 (12)	c.857G > A c.1168del	Missense Frameshift	Compound heterozygous	p.Arg286Gln p.Ala390Glnfs*6
3 (13)	c.875G > A c.1208G > A	Missense Missense	Compound heterozygous	p.Gly292Glu p.Arg403Gln
4 (14)	c.1577G > A	Missense	Homozygous	p.Arg526Gln
5 (11)	c.1792G > A	Missense	Homozygous	p.Arg561Gln
6 (8)	c.981 + 5G > A c.948C > T	Splice site Missense	Compound heterozygous	NA p.Arg280Cys
7a/7b (15)	*CLCN7*: c.594 + 193o*GNPTG*: c.178 + 6298	Nonsense	Homozygous	NA
8a/8b (30)	c.784_787del c.1354-1G > A	Frameshift Splice site	Compound heterozygous	p.Ser262Argfs*7NA
9 (9)	c.1504G > T	Missense	Homozygous	p.Arg502Trp
10 (3)	c.465_466ins	Frameshift	Homozygous	p.Tyr156Leufs*209
11 (3)	c.2338G > A	Missense	Homozygous	p.Arg767Trp
12 (3)	c.756G > A c.1614C > T	Missense Missense	Compound heterozygous	p.Gly240Arg p.Arg526Trp
13 (3)	c.1879T > C c.1485_1565del	Missense Frameshift	Compound heterozygous	p.Leu614Pro p.Tyr156Leufs*209
14 (3)	c.1158G > T c.*13621A > T	Nonsense Splice site	Compound heterozygous	p.Glu374* NA
15 (3)	c.1032A > G c.2337C > T	Missense Missense	Compound heterozygous	p.Met332Val p.Arg767Trp
16a/16b (3)	c.784C > G c.2269C > T	Missense Missense	Compound heterozygous	p.Pro249Arg p.Ser744Phe
17 (17)	c.1555 C > T c.2999 C > T	Missense Missense	Compound heterozygous	p.Leu519Phe p.Arg767Trp
18 (17)	c.286-9G > A c.1025T > C	Frameshift Missense	Compound heterozygous	p.Glu95Valfs*8 p.Leu342Pro
19 (18)	c.610A > T c.612C > G	Missense Missense	Compound heterozygous	p.Ser204Trp p.Ser204Trp
20 (19)	c.2416T > A	Nonsense	Homozygous	p.*806Argext*58
21 (16)	c.595-120_595-86dup	Splice site	Homozygous	NA
Present^a^	c.1208G > A c.982-1G > C	Missense Splice site	Compound heterozygous	p.Arg403Gln NA

NA, not applicable.

^a^ Current study.

In a review of 22 patients with *CLCN7*-related AROs, the predictive value concerning survival of early central nervous impairment was investigated (Table 2). Another important issue was that the onset symptom of exaggerated leukocytosis was scarcely reported, which would straightforwardly be misdiagnosed as JMML or LAD. However, the nonspecific peripheral blood smear test and the failure of bone marrow aspiration of the neonatal patient required the consideration of differential diagnoses. ARO is not recognized immediately in our case and other considerable number of children. Indicative signs, such as neurologic impairment, vision or hearing lost, and hematological abnormality, would be suggestive findings for the early diagnosis. Increased bone density is radiological feature for clinically early diagnosis. Also, high-throughput sequencing technology is the most effective and precise method to distinguish similar phenotypes. Hopefully, our research will be able to increase adequate knowledge and awareness of ARO disease for physicians, who might be the initial contact for patients, such as pediatricians, neurologists, hematologists, ophthalmologists, and geneticists. There are some limitations of our study. The minigene assay was an efficiency method to elucidate the biological effects of the novel splice site variant (c.982-1G > C). However, we did not perform studies on the working mechanism of this variant and more in-depth research is needed. Additionally, the predictive values of exaggerated leukocytosis as early signs need more clinical cohorts to illustrate.

## Conclusion

This study expands the spectrum of *CLCN7* mutations, reporting that the combination of c.1208G > A and c.982-1G > C mutant alleles resulted in a very early-onset, life-threatening phenotype. Furthermore, symptoms of leukocytosis, progressive neurological impairment, and vision or hearing loss should raise clinical suspicion of ARO. High-throughput sequencing technology is expected to be beneficial for precise diagnosis and improved prognosis.

## Data Availability

The original contributions presented in the study are included in the article/Supplementary Material, further inquiries can be directed to the corresponding author/s.
