# Supplementary material for: A novel compound heterozygous mutation of the CLCN7 gene is associated with autosomal recessive osteopetrosis
Source: Front Pediatr. 2023 Apr 24;11:978879. doi: 10.3389/fped.2023.978879 (PMC10165073; doi:10.3389/fped.2023.978879)
Supplement: Supplementary file 1 [file Datasheet1.docx]

Supplement Table 1:The PCR primers of two variants in *CLCN7* gene of the patient.

| **Variants** | **PCR Primers** | **Primer sequence** | **PCR product (bp)** |
| --- | --- | --- | --- |
| NM_001287.5: c.1208G>A | Forward | 5’-GGACACCTTTGCCCTGGAA-3’ | 405 |
|  | Reverse | 5’-CATACACAGCCTTTCTTTCGG-3’ |  |
| NM_001287.5: c.982-1G>C | Forward | 5’-TCCCCTCTTGCTCTCCACTG-3’ | 340 |
|  | Reverse | 5’-CTCCGAGTCAAACCTTCCGA-3’ |  |

PCR: Polymerase Chain Reaction

Supplement Table 2: Additional clinical biochemical investigation of the patient.

| **Item** | **Biochemical Value** | | **Ref range** |
| --- | --- | --- | --- |
|  | **Neonate** | **4 Months** |  |
| **Calcium-phosphorus Metabolism** | | | |
| **Calcium** | 1.87 | 2.52 | 2.1-2.55 (mmol/L) |
| **Phosphorus** | 2.2 | 1.83 | 0.81-1.45(mmol/L) |
| **PTH** | Null. | 4.94 | (pmol/L) |
| **Calcitonin** | Null. | 27.0 | (pg/mL) |
| **1, 25-(OH)-D3** | Null. | 30.83 | (ng/mL) |
| **ALP** | 196 | 132 | 38-126(U/L) |
| **LDH** | 786 | Null. | 106-211 (U/L) |
| **Immunoglobulin Levels** | | | |
| IgG | 10.2 | 6.97 | 7-16 (g/L) |
| IgA | <0.26 | 0.17 | 0.7-4.0 (g/L) |
| IgM | 0.32 | 1.60 | 0.4-2.3(g/L) |
| IgE | <4.56 | <4.23 | 0-100(IU/ml) |
| **Lymphocyte Classification Count** | | | |
| NK+ | 6.31 | 7.86 | 9.5-23.5% |
| CD3+ | 80.58 | 45.65 | 60.8-75.4% |
| CD3+/CD4+ | 61.77 | 27.65 | 29.4-45.8% |
| CD3+/CD8+ | 18.43 | 16.37 | 18.2-32.8% |
| CD3+/CD19+ | 10.75 | 45.25 | 6.8-15.8% |

PTH, parathyroid hormone; ALP, a lkaline phosphatase; LDH, lactate dehydrogenase;

NK: natural killer; +, positive;
